# Demystifying the link between higher education and liberal values: A within‐sibship analysis of British individuals’ attitudes from 1994–2020

**DOI:** 10.1111/1468-4446.12972

**Published:** 2022-08-28

**Authors:** Elizabeth Simon

**Affiliations:** ^1^ Department of Social Statistics and Demography University of Southampton Southampton UK

**Keywords:** higher education, liberal values, within‐sibship design

## Abstract

The link between university graduation and liberal values is well‐established and often taken as evidence that higher education participation causes attitudinal change. Identification of education’s causal influence in shaping individual preferences is notoriously difficult as it necessitates isolating education’s effect from self‐selection mechanisms. This study exploits the household structure of the Harmonized British Household Panel Study and Understanding Society data to tighten the bounds of causal inference in this area and ultimately, to provide a more robust estimate of the independent effect of university graduation on political attitudes. Results demonstrate that leveraging sibling fixed‐effects to control for family‐invariant pre‐adult experiences reduces the size of higher education’s effect on cultural attitudes by at least 70%, compared to conventional methods. Significantly, within‐sibship models show that obtaining higher education qualifications only has a small *direct causal effect* on British individuals’ adult attitudes, and that this effect is not always liberalizing. This has important implications for our understanding of the relationship between higher education and political values. Contrary to popular assumptions about education’s liberalizing role, this study demonstrates that the education‐political values linkage is largely spurious. It materializes predominately because those experiencing pre‐adult environments conducive to the formation of particular values disproportionately enroll at universities.

## 1 INTRODUCTION

Recent years have seen public discourse become increasingly critical of higher education (HE). Right‐leaning commentators have claimed, with increasing frequency, and ferocity, that professors at “woke” HE institutions “indoctrinate” students with “leftist agenda[s]” and “liberal madness” (Hopkins, 2016; Torres, 2020). Such assertions are grounded in research which has, since the 1950s, found with remarkable geographical and temporal consistency that people with higher levels of educational attainment, and particularly graduates, hold more liberal cultural views than their less educated counterparts (Weakliem, 2002). These findings have widely been interpreted as evidence that HE participation *causes* attitudinal change.

However, a growing body of literature authored by scholars including Campbell and Horowitz (2016), Kam and Palmer (2008), Persson (2014) and Sieben and De Graaf (2004) challenges this interpretation. It argues that research which controls only for measured pre‐adult characteristics will report biased effects of education on socio‐political orientations, as these variables, which are highly‐related to educational enrollment, are often measured poorly, or omitted, in survey data. As most existing empirical works utilize precisely this strategy, they tend to over‐estimate the magnitude, and statistical significance, of education’s effect on attitudes. This makes it difficult to ascertain whether the education‐liberal values associations reported in past research represent genuine causal, as opposed to spurious, effects. The question of whether HE study is the cause of graduates’ distinctive attitudes therefore remains an open one.

This paper applies a within‐sibship design to Harmonized British Household Panel Study (BHPS) and Understanding Society data (University of Essex, Institute for Social and Economic Research, 2021) from 1994 to 2020 to explore how HE participation shapes individuals’ economic, environmental and gender‐role attitudes. This data’s household structure affords a unique opportunity to better identify the causal effect of university graduation on attitudes. Exploiting the fact that siblings experience symmetrical pre‐adult environments, and modeling this within‐family invariance through fixed‐effect estimation, allows this study to go beyond the scope of existing works. Its central contribution is conducting a more robust empirical test of HE’s causal effect on British individuals’ attitudes and thus, advancing our knowledge of why graduates exhibit distinctive political values. Findings will not only indicate how the growth of HE might impact mass opinion, but also bring important empirical evidence to bear on claims, popular among right‐leaning commentators, that universities are hotbeds of left‐liberal bias.

## 2 HIGHER EDUCATION AND LIBERAL VALUES: UNDERSTANDING THE LINK

Decades of political socialization research has concluded that “political learning is a lifelong process, starting at an early age” (Neundorf and Smets, 2017, p. 3). Socio‐political attitudes are developed through encounters with socializing agents including families, peers, the media, education systems, and political and geographical contexts, in the “impressionable years” of childhood and early adulthood, and reach stability in adulthood (Alwin & Krosnick, 1991; Jennings & Niemi, 1981; Neundorf et al., 2013). However, these orientations are not then entirely fixed ‐ remaining subject to some degree of change throughout the adult life‐course (Miller & Sears, 1986). This greater malleability of attitudes in the “impressionable years” occurs as individuals more often experience environmental changes known to alter orientations, like entering HE or the workforce, leaving home or participating in social movements during early adulthood (Sears & Brown, 2013).

The association of educational attainment with socio‐political attitudes is one of the most robust social scientific findings (Weakliem, 2002). This well‐documented education effect has increasingly become regarded as a HE effect—with a plethora of studies demonstrating that the general education‐values linkage is predominantly driven by a marked graduate/non‐graduate attitudinal divide (Hainmueller & Hiscox, 2007; Stubager, 2008; Surridge, 2016). While it is evident that graduates typically possess a distinctive set of attitudes, less is known about whether HE participation, which constitutes one of the most profound environmental changes many individuals will experience during their life‐course, actually *causes* the development of these attitudes. This gap in knowledge largely stems from the fact that estimating education’s causal effect on political outcomes is fraught with methodological difficulty (Persson, 2014, 2015).

While it is possible that exposure to the educational content, or some other direct experiential aspect, of HE might lead students to develop distinctive political values,^1^ it seems equally plausible that this education effect is not causal but simply a proxy for other factors. Existing literature indicates that two key “proxy” education effects shape adult outcomes ‐ these are the pre‐adult socialization and sorting models (Persson, 2015). The former contends that, at least part of, the education‐liberal values association represents a self‐selection effect, as the same pre‐adult factors which shape attitudes—like intelligence, parental values and family socio‐economic status ‐ also determine educational attainment (e.g., Campbell & Horowitz, 2016; Sieben & De Graaf, 2004). In this view, the non‐random selection of individuals into universities, rather than any direct educational or experiential university effect, drives the education‐liberal values association.

The sorting model proposes it is the social position conferred upon us by virtue of being educated to a given level, rather than the experience of being educated, that determines adult attitudes (Stubager, 2008). Persons with high levels of educational attainment, and particularly graduates, typically achieve higher earnings, find more secure employment and occupy more central social network positions, than less educated persons (Bovens & Wille, 2017). For proponents of the sorting model, attitudinal asymmetry between educational groups is driven by these stratification‐based experiences. Graduates might, for example, be more supportive of immigration and tolerant toward ethnic minorities, than non‐graduates, because they are less exposed to job competition from low‐skilled migrant workers (Hainmueller & Hiscox, 2007) or because they are more often embedded in networks endorsing these attitudes.

Clearly then, estimating education’s causal effect on attitudes necessitates fully isolating education’s influence from the highly related effects of pre‐adult, and stratification‐based, experiences. Existing studies have typically sought to do this by using multiple regression techniques to estimate education’s effect on attitudes, net of controls for these confounders (see Dey, 1996; Hainmueller & Hiscox, 2007; Paterson, 2009, 2014; Phelan et al., 1995; Stubager, 2008; Surridge, 2016; Van De Werfhorst & De Graaf, 2004). Though conducted across several decades, and various advanced Western democratic contexts, these studies’ conclusions are remarkably consistent. Generally, they find that even after controls for education‐as‐a‐proxy explanations, persons with high educational attainment, and particularly graduates, exhibit considerably more liberal cultural attitudes and somewhat less liberal economic attitudes,^2^ than their less educated counterparts.

While this field of enquiry has advanced our knowledge of the education‐liberal values linkage ‐ by demonstrating that some educational or experiential aspect of HE study *is related to* developing distinctive attitudinal profiles—the extent to which this education effect is *causal* remains unclear. This is because causal conclusions cannot be inferred from “conventional” regression‐based studies, which only identify associations. An emerging line of research makes this point clearly. It contends that because surveys inevitably cannot collect data on all important pre‐adult characteristics, and often measure those collected with error, education’s effect on adult outcomes will be over‐estimated in observational studies which control only for measured pre‐adult characteristics, as effects will remain subject to further confounding influences (Campbell & Horowitz, 2016; Kam & Palmer, 2008; Persson, 2014; Sieben & De Graaf, 2004). This makes it impossible to ascertain whether the education‐liberal values associations reported in past research represent genuine causal, as opposed to spurious, effects.

To tackle this causal identification issue, pioneering studies have used sophisticated quantitative techniques including regression discontinuity, fixed and random‐effect and matched designs which control for unmeasured and poorly measured (as well as measured) pre‐adult characteristics. This body of work has explored education’s *causal* effect on two broad types of adult outcomes: political engagement—including participation, citizenship and voting (Kam & Palmer, 2008; Marshall, 2016; Mayer, 2011; Perrin & Gillis, 2019; Persson, 2014) ‐ and socio‐political attitudes (Campbell & Horowitz, 2016; Kunst, Kuhn, and van de Werfhorst, 2020; Scott, 2022; Sieben & De Graaf, 2004). While attitudinal research has typically found the strong and statistically significant education effects reported in “conventional” studies are greatly reduced in size, and sometimes entirely nullified, under more robust testing, the picture has been more mixed in studies of political engagement. Marshall (2016), Mayer (2011) and Perrin and Gillis (2019), for example, all find education has a *substantial causal effect* on political engagement, even after controls for spurious effects. Nevertheless, these studies all demonstrate that “conventional” regression designs considerably over‐estimate education’s “true” effects and thus, that the methodological limitations of past research may have prevented us gaining an accurate understanding of how precisely education shapes political outcomes.

Despite substantial advances in our knowledge of how best to obtain unbiased estimates of education’s effect on adult outcomes, and thus tighten the bounds of causal inference in this area, few studies have put these into practice. As yet, only a single study conducted in the British context (see Scott, 2022) has employed this kind of sophisticated quantitative technique to identify education’s independent effect on liberal values. Therefore, it remains a relatively open question as to whether the association of HE and liberal socio‐political attitudes, observed in Britain, is genuinely causal. This study seeks to advance our knowledge of the mechanisms driving this well‐established association, by asking: *does studying for a degree cause British graduates to develop distinctive (il)liberal economic and cultural attitudes?* It is hypothesized that the effect of HE on attitudes reported using “conventional” methods will reduce in size, and potentially be nullified, when estimated under more stringent tests of causality.

### 2.1 The British case

Britain is an interesting case for exploration, not only because education has been a major driver of attitude formation and political behavior, in this context, in recent years (Ford et al., 2021; Simon, 2021), but because it is likely to become increasingly so in the future—as decades of educational expansion alter the educational composition of the population (Sobolewska & Ford, 2020). Exploring this study’s research question in the British context will not only provide novel evidence regarding whether HE plays an un‐anticipated societal function, in shaping graduate attitudes, but will also have important wider implications. Firstly, it will provide insight into how expanding HE enrollment, and growth in the graduate population, may alter aggregate British public opinion. Secondly, it will allow assessment of the validity of claims, popular among right‐wing commentators, that universities are centers of left‐liberal indoctrination. Given that educational polarization threatens societal cohesion and the functioning of British democracy, evidence about the nature of the education‐liberal values association is needed now more than ever.

## 3 DATA

This study draws on high‐quality, nationally representative data from the BHPS and Understanding Society surveys to quantify the effect of graduation on socio‐political attitudes, net of the confounding influences of pre‐adult characteristics and adult stratification‐based experiences. It exploits that data collection from eligible BHPS respondents (1991–2008) continued from 2009 as part of Understanding Society (Fumagalli et al., 2017) to conduct longitudinal analysis spanning over 30 years.

The combined panel dataset contains data collected at 28 annual intervals from all individuals aged 16 and over residing in originally sampled BHPS households, and all those who subsequently came of age, or to reside with original sample members (Fumagalli et al., 2017). By virtue of its’ longitudinal household design, this data not only contains repeated measures of individual‐level variables, including educational attainment, socio‐demographics, and attitudes, but also provides details of how respondents are related and therefore facilitates matching of individuals within households. This allows the shared backgrounds of siblings to be leveraged in analyzing education’s effect on attitudes, and thus for both the unmeasured and measured pre‐adult characteristics which confound this relationship to be controlled. Such detailed information affords a unique opportunity to better identify education’s causal effect on attitudes.

While this longitudinal dataset is ideal for exploring the extent to which education fosters value change, versus value reinforcement due to self‐selection (Surridge, 2016), as it allows pre‐adult and adult environments to be controlled when estimating attitudinal change over a period, doing so requires that response attrition is addressed. Attrition is problematic for two reasons. Firstly, it reduces the size of samples for analysis and therefore the precision of estimates. Secondly, because if attrition is selective (the characteristics of (non‐)respondents vary) inferences drawn from analyses will not be generalizable to the intended target population. As decades of study into attrition in large‐scale panel studies has generally concluded this “rarely seems to introduce substantively important bias” (Lynn et al., 2005, p. 20), the former issue is the primary concern.

### 3.1 Measurement intervals

To avoid reduction in sample size the indicators constructed utilize multiple waves of data. Generally, adult measures are recorded for graduates in the wave they first report obtaining a HE qualification, or in any of the nine subsequent waves, if this information is missing at earlier waves. Because socio‐political attitudes are associated with age (Peterson et al., 2020), and there is a strong age‐gradient in educational attainment (Sobolewska & Ford, 2020), non‐graduates’ adult attitudes needed to be measured so as to ensure reported educational differences in attitudes were not a product of the groups’ varying age profiles. As exploratory analysis revealed the median age of sampled graduates was 23^3^ (Appendix A), non‐graduates’ attitudes were recorded in the wave they turned 23, or at any of the nine subsequent waves ‐ mirroring the graduate adult data collection window. Although data collection windows used for pre‐adult characteristics vary, all use multiple waves to maximize response, and collect data no later than when respondents were aged 20, or three waves prior to HE graduation. This collection period reflects that adult reporting begins at 23, and obtaining a degree requires at least three years full‐time study. Table 1 provides details of all variables, data collection periods and valid responses.

**TABLE 1:** Operationalization, measurement and valid observations

Classification		Variable	Data collection window	Observations
Dependent variables		Adult gender attitude scale	0–9 wave(s) post‐graduation or ages 23–31, for non‐graduates	11,048
		Adult economic attitude scale		3108
		Adult environmentalism scale		7353
Key independent variable		HE graduation status	First report	59,942
Control variables	Socio‐demographic variables	Gender		
		Cognitive ability		
		Psychological security		
		Occupational class	0–9 wave(s) post‐graduation or ages 23–31 (non‐graduates)	15,449
	Pre‐adult characteristics: family variant	Membership of community groups	Age 20 or younger	59,942
		Membership of sports groups		
		Participation in cultural activities		
		Birth order	Derived for sibling respondents only using first report of birth year	38,802
	Pre‐adult characteristics: early attitudes	Pre‐adult gender attitude scale	3–12 waves pre‐graduation or age 20 or younger (non‐graduates)	9597
		Pre‐adult economic attitude scale		2274
		Pre‐adult environmentalism scale		6103
	Pre‐adult characteristics: Family invariant	Parental occupation	Reported by parent when respondent was aged 20 or younger	10,879
		Parental education		10,768
		Parental income		10,926
		Parental engagement in parent‐teacher associations (PTA)		59,942
		Parental gender attitude scale		6305
		Parental economic attitude scale		1369
		Parental environmentalism scale		3829

*Note*: Those with missing educational information are excluded.

While it would have been preferable to account for the differing lengths of time taken by full‐ and part‐time students to graduate, by varying the time elapsed between (pre‐)adult reporting windows, a 3‐year study duration was assumed for all graduates, as the data contained no reliable, or consistent, indicator of study mode. While this means pre‐adult measures for part‐time students will have been recorded in the early stages of their undergraduate study, and therefore renders controls for spurious effects somewhat imperfect, it is not believed this will significantly bias conclusions drawn. Firstly, because part‐time students make up just 15% of UK undergraduates (Hubble & Bolton, 2021), and secondly, because Mintz (1998, p. 32) indicates that just “2 years of university education is insufficient to have a discernible effect on [the attitudes of] young adults”. Nevertheless, a sensitivity analysis was conducted to explore whether conclusions about HE’s effect on attitudes varied depending on the gap between reporting pre‐adult and adult measures. Results presented in Appendix B show 3‐ and 4‐year reporting periods yield similar estimates. Subsequent analyses use the 3‐year specification, as larger sample sizes afford greater statistical power.

### 3.2 Dependent Variable(s)—Liberal values

This study uses three attitudinal dependent variables which measure adult attitudes toward gender‐roles, the economy, and the environment. These capture respondents’ positions on the two core ideological dimensions which define the UK’s political space ‐ the economic, or left‐right, dimension and the cultural, or new politics, dimension (Evans et al., 1996). The economic attitudinal measure corresponds to the former dimension and covers issues of inequality, exploitation, and government regulation. The other measures capture the cultural dimension, which encompasses “issues concerned with lifestyle, ecology, cultural diversty, nationalism and immigration” (Hooghe et al., 2002, p. 976).

Multi‐item indicators measure attitudinal positions with considerably less error than individual survey items, and produce measures which are highly stable over time (Ansolabehere et al., 2008). Therefore, each attitudinal measure used comprises a multi‐item scale, constructed by averaging Likert response‐scale items across the three issue areas. All individual attitudinal items included had five response categories representing: strong agreement, some agreement, neutrality, some disagreement and strong disagreement. Responses were coded so high values indicated “liberal” positions: left‐wing economic attitudes, environmental concern, and support for gender egalitarianism. Explanatory factor analysis ensured all items selected for each measure captured the same, single attitudinal dimension. All items contributing a rotated factor loading exceeding 0.2 on factors with eigenvalues greater than one were selected for use.

**FIGURE 1:**
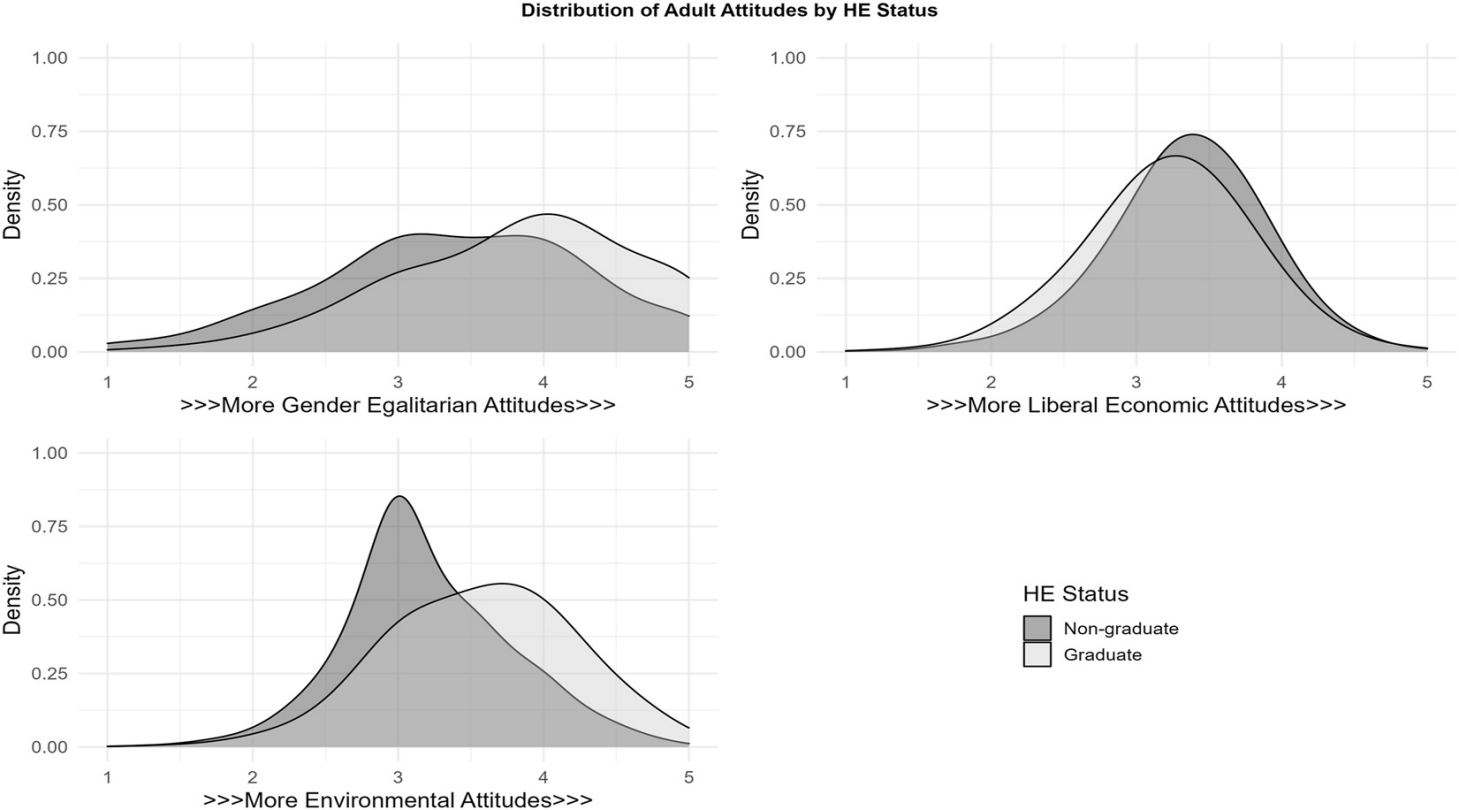
Educational distribution of dependent attitudinal variables

As slightly different batteries of questions on environmentalism were used in the BHPS and Understanding Society data, this process yielded four separate scales—two for environmentalism, and one each for economic and gender attitudes. These separate environmentalism scales were combined. Although this means inferences drawn about one of the three attitudinal outcomes relies on responses measured across subtly different scales, combining these was necessary to preserve sample size, and should not prove problematic given preliminary analysis showed these scales’ distributions were near identical (Appendix C). Survey items shown to load sufficiently on each attitudinal dimension in factor analysis were summed together and divided by the total number of scale items. This produced three attitudinal scales running from 1–5, with higher values indicating greater liberalism. Tests indicated these scales were, in general, highly reliable.^4^ The educational distribution of each attitudinal dependent variable is shown in Figure 1 and Appendix D details the items included in each scale.

### 3.3 Key independent variable—HE status

This study aims to ascertain whether HE, specifically, rather than exposure to increased levels of education engenders attitude change. A simple binary measure of educational attainment, which indicates whether respondents have HE qualifications, or not, is therefore appropriate. In the UK, HE typically refers to educational courses undertaken after school leaving. This study uses a more stringent definition, which classifies only those who have achieved at least a Bachelor’s degree, or equivalent, as HE graduates.

15,162 of the 75,104 sample respondents either reported obtaining a degree prior to joining the panel, or prior to being a respondent for three years. These respondents were excluded from analysis as, assuming a 3‐year minimum study duration, it would have been impossible to observe their pre‐HE attitudes ‐ which must be controlled to draw accurate inferences about educations’ impact on attitudes. 2650 of the remaining 59,942 respondents were classified as graduates and 57,292 as non‐graduates. As respondents were allocated to the “graduate” condition after obtaining their first university qualification, and the UK’s educational system typically requires an undergraduate degree for progression to postgraduate study, it can be assumed that the HE effect captured pertains to gaining an undergraduate degree.

### 3.4 Control variables

Table 1 shows all variables used in the analysis (Appendix E contains full details of coding and descriptive statistics). All socio‐demographic and pre‐adult variables are controls—included either to account for individual differences in attitudes and educational attainment, or as they are known to confound the HE‐adult attitudes association. Accounting for these variables ensures spurious and causal education effects are separated in estimation. While all pre‐adult variables serve the same purpose statistically, conceptually they can be sub‐divided into three categories: respondents’ early attitudes, pre‐adult characteristics which vary within families and family‐invariant pre‐adult characteristics, which are effectively measured at the household‐level.

All controls, except occupational class, are reported pre‐adulthood and designed to capture spurious effects associated with self‐selection. Occupational class is reported in adulthood and intended to capture spurious effects linked to stratification‐based sorting mechanisms, by acting as a proxy for respondents’ labor market situation—for example, income, economic security, and prospects of economic advancement. The fact that adult attitudes are reported up to 9 years post‐HE graduation here makes it essential that adult status was controlled. Without such controls, it would have been impossible to ascertain whether it was HE study itself or post‐HE stratification‐based experiences driving any attitudinal change reported. Given that education opens the door for occupations, this study should be seen as providing a more robust estimate of HE’s *direct causal effect* on attitude formation, rather than its *total causal effect*.

Cognitive ability is a composite measure, capturing both literacy and numeracy skills. Literacy is measured using verbal fluency testing which scores respondents on the number of items per category (e.g., “animal”) they can name in 1 minute. Numeracy is measured using “number series” tests which score respondents’ ability to identify missing numbers from sequences. Literacy and numeracy scores were standardized, averaged, summed and this measure then divided into three equally sized groups to demarcate high, medium and low ability. As cognitive ability was only measured in wave 3 of Understanding Society, this variable had a high proportion of missing responses. To prevent loss of sample size, this indicator includes a “no information” category. While it would have been desirable to include separate literacy and numeracy measures, this was not possible due to collinearity—almost all individuals with no numeracy information also had none for literacy. Psychological security is measured using the statement “I feel that what happens in life is often determined by factors beyond my control”. Responses were coded into three categories, representing those who: agreed, disagreed, and did not provide information. The latter was used as this measure, like the previous, draws on responses from a single wave.^5^

Studies have shown birth‐order influences our pre‐adult environment, with first‐born children, for example, spending more quality time with their parents (Price, 2008). A dummy variable which reports whether sibling respondents were the first‐born in their household, or otherwise, is therefore included to control for the fact that even siblings raised in the same household can experience different family environments.^6^ Doing so ensures maximum symmetry in siblings’ pre‐adult environments and thus, improves this study’s ability to identify causal HE effects.

Pre‐adult and parental attitude scales were coded identically to the adult attitude scales. The only differences between these variables is that they are recorded at different occasions and by different persons—either by the respondent themselves, or by their parent(s) or guardian(s). Where respondents’ mother and father both provided valid responses for an attitudinal scale, their scores were averaged to create a combined parental attitude measure for that dimension.

All family‐invariant pre‐adult characteristics are reported by respondents’ parents. This prevents measurement error associated with proxy reporting leading to over‐estimation of education’s effect on attitudes (Sieben & De Graaf, 2004). Parental income, education, occupation and PTA membership are categorical variables, coded to represent the highest value reported between the respondents’ mother and father, or their mothers’/fathers’ value, if only one parent is identified, and set to missing, if no parental information was available.

## 4 ANALYTICAL STRATEGY

This study’s research question is answered by examining results obtained from a series of three, sequentially‐built ordinary least squares regression models—one for each dependent attitudinal variable. The model building sequence is outlined in Table 2.

**TABLE 2:** Model building

Variables included	(1) Education only	(2) Sibling‐education only	(3) Education and self‐selection	(4) Education, self‐selection and pre‐adult attitudes	(5) Sibling ‐ matched
Dependent variable	Adult attitude				
Independent variable(s)	HE status		HE status, socio‐demographics and all pre‐adult characteristics, **except** early attitudes and birth order	HE status, socio‐demographics and all pre‐adult characteristics **except** birth order^7^	HE status, socio‐demographics, family variant pre‐adult characteristics, pre‐adult attitudes and birth order **plus** sibling fixed‐effects

Block 1 reports the “raw” HE‐liberal values association for the full sample. This model provides a benchmark against which the reduction in the size of HE’s effect on attitudes engendered by including various controls, in blocks 3, 4 and 5, can be measured. As there may be limited overlap between respondents in the full and sibling‐only samples, it is possible that differences in HE effects estimated between model blocks 1, 3 and 4 and block 5 could be attributed to selection bias, rather than genuine changes engendered by sibling fixed‐effects. Block 2 models, which report the “raw” HE‐liberal values association for the sibling‐only sample were estimated to explore this possibility. Blocks 3 and 4 replicate “conventional” analytical strategies employed in existing works—showing the effect of HE on attitudes, after isolating this from measured selection‐into education and stratification effects. Block 5 models go beyond the scope of existing analyses by adopting within‐sibship designs which simultaneously control for unmeasured and measured aspects of pre‐adult experience and therefore, improve our ability to identify HE participation’s *direct causal effect* on attitudes.

### 4.1 Sibling matching

Following the approach of Campbell and Horowitz (2016), restrictions were imposed to ensure siblings had experienced similar early socialization environments, and thus that within‐sibship estimation would provide the fullest controls for spurious education effects. Therefore, sibling clusters were created by matching only sibling respondents (natural, half‐, step‐, adopted and foster siblings) who had reported living together, in the same household, at the first wave in which they were surveyed. Before accounting for missing data, this process of sibling matching yielded a sub‐sample of 16,093 sibling clusters representing 38,802 individual respondents. While most of these sibling clusters were comprised of just 2 siblings (73% of clusters), around a quarter of all sibling clusters formed ranged from sizes 3 to 5 (inclusive), and just over 1% of sibling clusters included 6 or more siblings. This sibling sample was somewhat more educated, and more active in community, sporting and cultural activities pre‐adulthood, than the full sample (Appendix F).

These sibling clusters not only serve as the unit of analysis in sibling fixed‐effects models (models 2 and 5, see Table 2), but also form the basis of the sibling cluster‐robust standard errors which are calculated across all models, to ensure variance estimates are not biased on account of the fact siblings are likely more similar than unrelated individuals (Cameron & Miller, 2015).

## 5 RESULTS

**FIGURE 2:**
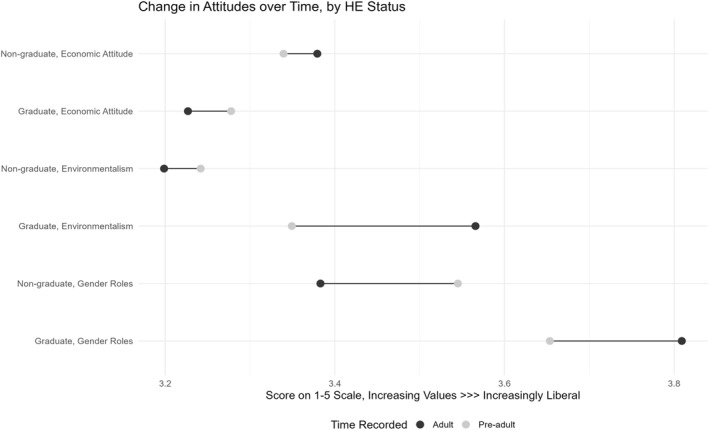
Change in graduates’ and non‐graduates’ attitudes over time

Preliminary analyses explored the average pre‐adult and adult attitudinal positions of graduates and non‐graduates in the full sample, to ascertain the extent, and direction, of any attitudinal change experienced. Figure 2 plots these statistics and shows that graduates’ attitudes do, on average, change over this period, and often do so more dramatically than, or in the opposite direction to, non‐graduates’. Two sample *t*‐tests confirm all educational differences in attitudes observed in adulthood are statistically significant at the 1% level (Appendix G). This evidence clearly indicates it is plausible that British graduates could develop their distinctively (il)liberal attitudes as a direct product of HE participation.

**FIGURE 3:**
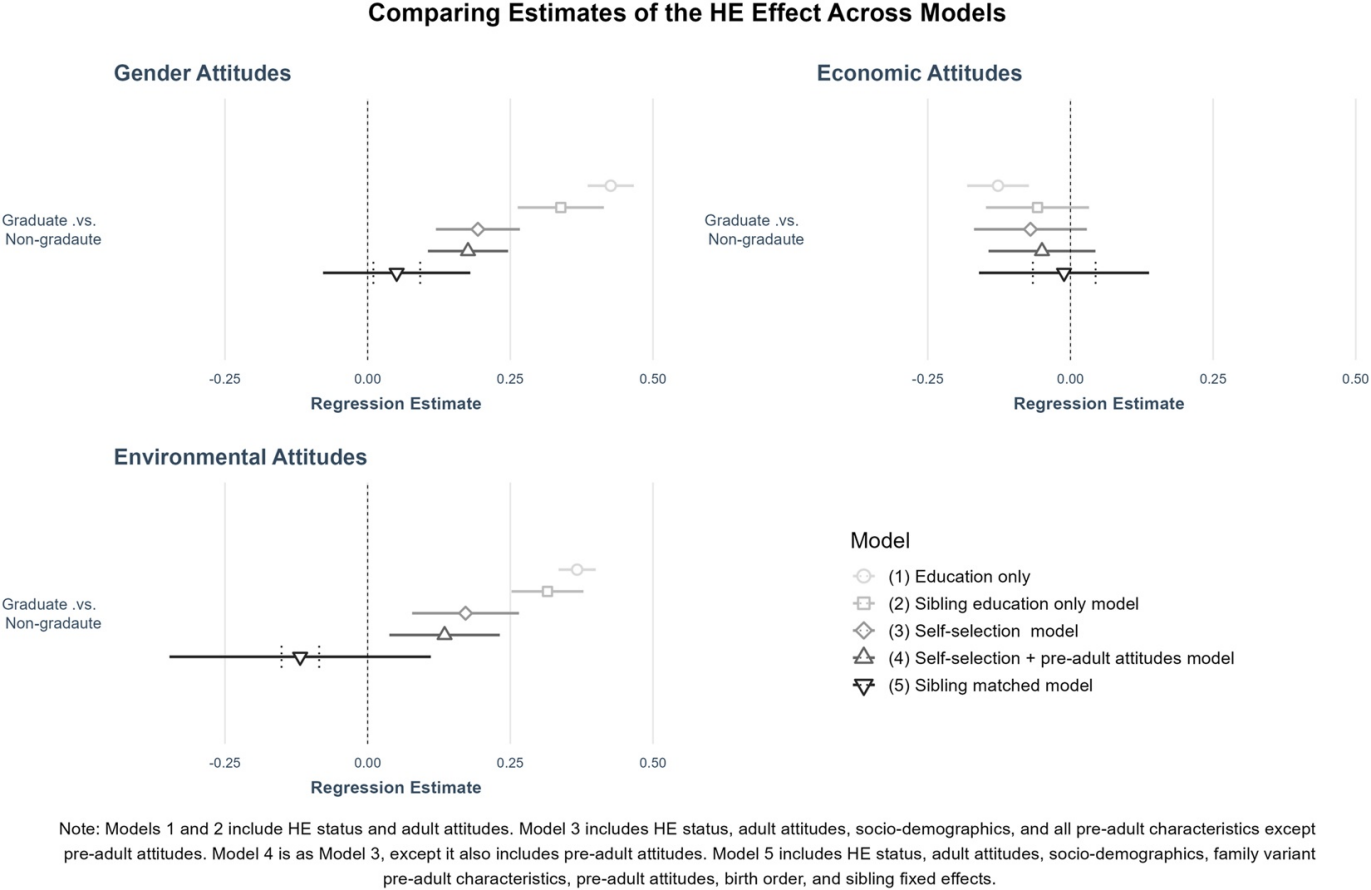
Plot of educational coefficients estimated in regression models

The remainder of this section presents regression results. Table 3 and Figure 3 illustrate how educational coefficients reported across the three attitudinal regression models estimated change with variable additions (blocks 3, 4 and 5), and show the raw education‐attitude associations for the full and sibling‐only samples (blocks 1 and 2). They present coefficients of the education variable only, as these are the primary statistics of interest in answering the research question (Appendix H presents full regression results).

**TABLE 3:** Educational coefficients estimated in regression models

	(1) Education only	(2) Sibling ‐ education only	(3) Self‐selection	(4) Self‐selection and pre‐adult attitudes	(5) Sibling‐matched
Gender attitudes					
HE status: Graduate	0.426***	0.338***	0.193***	0.176***	0.051
	(0.021)	(0.039)	(0.038)	(0.036)	(0.065)
Observations	11,048	2240	2296	2171	1278
Economic attitudes					
HE status: Graduate	−0.127***	−0.058	−0.070	−0.050	−0.011
	(0.028)	(0.046)	(0.050)	(0.048)	(0.076)
Observations	3108	569	652	616	375
Environmental attitudes					
HE status: Graduate	0.367***	0.315***	0.172***	0.135***	−0.118
	(0.017)	(0.032)	(0.048)	(0.049)	(0.116)
Observations	7353	1769	869	746	268

*Note*: Regression coefficients are presented with sibling‐clustered standard errors in parentheses. Models 1 and 2 include HE status and adult attitudes. Model 3 includes HE status, adult attitudes, socio‐demographics, and all pre‐adult characteristics except pre‐adult attitudes. Model 4 is as Model 3, except it also includes pre‐adult attitudes. Model 5 includes HE status, adult attitudes, socio‐demographics, family variant pre‐adult characteristics, pre‐adult attitudes, birth order, and sibling fixed effects.

Significance is denoted by ***p < 0.01; **p < 0.05; *p < 0.1.

Block 1 results indicate that adult gender, economic and environmental attitudes are all associated with HE status, in the full sample (see Table 3). Apart from economic attitudes, where graduates take a statistically significantly more conservative position than non‐graduates, graduates typically have attitudes which are significantly more “liberal” than non‐graduates—they are more environmentally friendly and gender egalitarian. The strongest “raw” educational effect observed is for gender‐role attitudes, where graduates report being 0.426 points more liberal than non‐graduates. The smallest effect is for economic attitudes. Graduates are typically only 0.127 points less economically liberal than non‐graduates.

Comparing block 1 and 2 estimates of HE’s effect on adult attitudes highlights that while the magnitude of the “raw” education‐attitudes associations reported are generally smaller in the sibling‐only samples, they are broadly similar to those reported for the full samples. This is true of all attitudinal models except the economic attitudes regression, where not only is the HE effect reported in the sibling sample (model 2) less than half the size of that in the full sample (model 1), but this effect ceases to statistically significant in the sibling model. This disparity likely stems from differences in the socio‐demographic composition, and pre‐adult experiences, of the samples (Appendix F), and suggests that the sibling‐only sample used to estimate the economic attitudes model is somewhat un‐representative of the wider sample. Some caution should therefore be expressed when interpreting the results of sibling‐only economic attitudes regressions.

The economic attitudes regressions exhibit a different pattern to the cultural attitudes regressions. As soon as even the least stringent controls for spurious education effects are introduced in block 3, the effect of HE on adult economic attitudes is not only dramatically reduced in size (45% smaller than in block 1) but ceases to be statistically significant. Effect sizes reported for gaining a HE qualification, compared to not doing so, are tiny, net of controls—estimated to shift economic attitudes by considerably less than even one tenth of a scale point in the conservative direction, in the block with the largest educational coefficient (block 3). These results provide evidence to suggest studying at university is not the cause of British graduates’ distinctively (il)liberal economic attitudes. Rather, self‐selection and stratification‐based sorting is at play. Differences in graduates’ and non‐graduates’ economic attitudes in Britain are a product of these groups’ typically divergent early life and adult status experiences.

In both cultural attitudes models the magnitude of the HE effects estimated in blocks 3, 4 and 5 are considerably smaller than the “raw” education effects (block 1 and 2), and their size reduces in a linear pattern across blocks 3, 4 and 5 (see Table 3). Figure 3 makes this point clearly—showing that as each subsequent block introduces more stringent controls for spurious education effects, the education coefficients in cultural attitudes models shrink toward zero (and in one case even become negative). These patterns were expected and suggest controls for proxy effects work as intended. Only once the most stringent controls for self‐selection and sorting—the sibling fixed‐effects—were introduced to the gender and environmental attitude models did the reported HE effects attenuate sufficiently to become non‐significant, at the 5% threshold. The reductions in the size of HE effects engendered by sibling fixed‐effects were substantial. In block 5 models the positive, “liberalizing” effect of HE attendance is completely eradicated, and replaced by a small negative effect, for environmental attitudes, and is reduced in size by 71% (compared to the conventional model with the fullest controls for spurious effects ‐ block 4) in the gender attitudes model.

It should be noted that although the large reductions in effect magnitude observed between block 4 and block 5 estimates (see Figure 3) seem to indicate clearly that sibling fixed‐effect models provide considerably less biased estimates of education’s causal effect on adult cultural attitudes than conventional models, the overlap of these estimates’ 95% confidence intervals means we cannot be completely certain this is the case.

Taken at face value, the null block 5 results presented in the gender attitudes and environmentalism models could be interpreted as evidence that HE study *does not cause* British graduates to develop distinctively liberal cultural attitudes. With it being argued that these results instead show that the association of HE participation with liberal cultural attitudes is spurious, driven largely by the fact that those experiencing pre‐adult environments which encourage the formation of particular attitudinal profiles disproportionately go on to obtain HE qualifications. However, some further consideration is required.

Sibling fixed‐effects models are power‐hungry relative to conventional models—with estimates in sibling models being less precise than full sample estimates, due to the relatively smaller size of sibling samples. Judgments must therefore be made about whether null within‐sibship findings indicate a genuine absence of causal effect, or simply that estimates are too imprecize to be conclusive (Madsen et al., 2014). This involves considering the extent to which the loss of significance in the HE effects seen in the sibling fixed‐effect (block 5) cultural attitudes models comes from attenuating effect sizes, as opposed to larger standard errors.^8^ To aid in making this decision, 95% confidence intervals equal to the width of those estimated for the block 1 education effects, which have the largest sample size, were superimposed onto the block 5 effect ‐ see the dashed lines in Figure 3.

None of the simulated confidence bounds overlap zero for the cultural attitude models—indicating that the within‐sibship estimates of the HE effect *would have* been statistically significant if estimated in larger samples. The null HE effects in Table 3 and Figure 3, are at least in part, a product of the loss of power engendered by sibling fixed‐effect estimation. This suggests a more tentative reading of this study’s results is appropriate. Spurious self‐selection and stratification‐based sorting mechanisms are not the sole drivers of the association between HE study and cultural attitudes. Rather, in Britain, graduating from HE has a small liberalizing effect on gender‐role attitudes, and a small inverse effect on environmentalism—with HE attendees becoming slightly less environmentally friendly, relative to non‐attendees, during their studies. Ultimately, this analysis suggests firstly, that university study has a small *direct causal effect* on adult cultural attitudes in Britain, and secondly, that these effects are not always liberalizing.

It is important to put the size of these HE effects in context. Consider that even the largest *liberalizing* HE effect on adult attitudes reported in the within‐sibship (block 5) models is 0.051, for the gender attitudes outcome. Put this way, this study finds that obtaining a HE qualification *causes* graduates to become just over one 20th of a scale point more culturally liberal than those who do not attend HE. Given all attitudinal scales run from 1–5, it seems fair to say that although HE does indeed have a small *direct causal liberalizing effect* on gender attitudes, it is unlikely that such subtle liberalizing effects will have any dramatic impact on aggregate British public opinion.

## 6 CONCLUSION

This paper goes beyond the scope of existing work by conducting a more robust test of the independent effect of HE participation on political values, through use of a within‐sibship design which tightens the bounds of causal inference. In doing so, it advances our understanding of the mechanisms driving this association—revealing that university graduation itself only has a small *direct causal effect* on British individuals’ adult attitudes. This finding holds irrespective of the duration between pre‐adult and adult measurement used. The remainder of this section discusses the crucial implications of this study’s findings both within, and beyond, academia.

Firstly, and perhaps most interestingly, this study provides evidence to suggest that studying at university only has a modest *direct causal effect* on British graduates’ attitudes and, importantly, that this effect is only liberalizing in the case of gender‐role attitudes—HE attendees actually develop slightly more conservative economic and environmental adult attitudes, relative to non‐attendees. In doing so, this study finds limited evidence that HE participation *causes* graduates to develop distinctively liberal political values. Rather, it highlights that self‐selection and stratification‐based sorting are the key drivers of the British education‐liberal values linkage. Differences between graduates’ and non‐graduates’ attitudes materialize predominantly because individuals with pre‐adult experiences predisposing them to develop particular attitudes disproportionately go on to obtain degrees. The implication of this finding is important. Right‐leaning commentators’ claims that universities are hotbeds of left‐liberal bias are greatly exaggerated—at least in this national context.

It is worth noting that, due to data deficiencies, this study could not account for respondents’ social networks—which Persson (2015) argues are an essential mechanism through which “sorting” effects operate ‐ when estimating the association of HE with adult attitudes. It is therefore quite plausible that the only (modest) “liberalizing” effect of HE study on attitudes detected in this study (for gender egalitarianism) could be driven by interactions amongst peer networks on university campuses, rather than by a top‐down process of “indoctrination” engendered by professors or the official curriculum. This further supports this study’s refutation of the “indoctrination hypothesis”—suggesting that even the modest *direct causal HE effect(s)* on attitudes identified here cannot straightforwardly be attributed to formal processes of socialization experienced at university.

Secondly, this study finds that using a within‐sibship design, which controls for unmeasured family‐invariant pre‐adult experiences in addition to measurable pre‐adult experiences and adult status indicators, likely provides considerably less biased estimates of HE’s effect on adult cultural attitudes than “conventional” methods. While this finding was expected, and mirrors previous conclusions drawn by Campbell and Horowitz (2016) and Sieben and De Graaf (2004) in US and Dutch contexts, the magnitude of bias reduction was striking. HE effects were at least 70% smaller in within‐sibship cultural attitudinal models, than in conventional models. This paper provides persuasive evidence which contributes to a growing consensus that quasi‐experimental designs must be employed if accurate estimates of education’s effect on attitudes are to be produced. Future works must make wider use of household panel studies, which allow use of within‐sibship designs, when seeking to identify causal effects.

This study finds HE graduation has considerably less substantial effect(s) on British individuals’ adult attitudes than “conventional” analyses—see Paterson (2009, 2014) and Surridge (2016). This disparity is unsurprizing and highly likely a product of methodological differences—this study found a relatively smaller effect of HE on adult attitudes as it leveraged high‐quality, household structured, longitudinal BHPS and Understanding Society data to perform a more robust test of the association of interest. Encouragingly, this study’s findings are broadly comparable with those presented by Scott (2022) in a recent analysis which uses sophisticated quantitative techniques to isolate spurious and causal HE effects, in Britain. While both analyses show HE graduation *causes* more egalitarian (liberal) attitudes (although this study considers gender egalitarianism, rather than racial prejudice), it is worth noting that the causal HE effect reported by Scott is somewhat larger in magnitude than that presented here. There are several explanations for this. Firstly, different attitudinal measures are used across these studies, and secondly, Scott (2022, p. 8) reports the *total causal effect*, rather than the *direct causal effect,* of HE on attitude formation, and so includes within this estimate “downstream effects such as differences in socio‐economic position” which are omitted here.

It is also possible that differences in the conclusions drawn by this study, and the works of Paterson (2009, 2014), Scott (2022) and Surridge (2016) are, at least to some extent, a product of the varying temporal contexts in which these studies were conducted. These existing British analyses largely rely on the British Cohort studies, and thus, explore the HE effect amongst pre‐2000 graduates.^9^ This study’s HE effect reflects that of a later period (graduating from 1994 to 2020). This variation in timing is important as government emphasis on social mobility drove a rapid period of HE expansion during the 1990s (Boliver, 2011). This fundamentally transformed the UK HE sector. New institutions and types of course were created, funding arrangements were altered, and campuses became more diverse spaces, as enrollment of “non‐traditional” HE entrants was encouraged (Bathmaker, 2003; Carpentier, 2018). As a result, studying at university today marks a qualitatively different experience to what it did just two decades ago. It therefore seems possible that the effect of HE participation on British graduates’ adult attitudes would vary across cohorts enrolling during different stages of the sectors’ development. Acknowledging this not only underlines the importance of not generalizing this studies’ findings beyond the cohort of graduates to which it applies, but highlights that we cannot simply assume differences in the conclusions drawn by this study, and earlier British studies, are linked purely to methodological differences. Future research should endeavor to disentangle the relative contribution of cohort and methods‐based influences to these disparities, for example, by leveraging twin data from the British Cohort studies to re‐examine Paterson’s (2009, 2014), Scott’s (2022) and Surridge’s (2016) findings.

The novel findings presented in this paper have crucial implications for scholars of social science. Advancing our knowledge of the mechanisms driving the association of education with liberal values—by showing that British graduates’ distinctive adult attitudes are only to a small extent a *direct consequence* of university study, and rather are largely determined by their distinctive pre‐adult, and adult status, experiences ‐ provides a better idea of what educational attainment, which is one of the most commonly used controls in social science research (Persson, 2015), is actually controlling for when added to analyses. In doing so, this study facilitates the making of better theoretically informed decisions in model‐building across future projects. These findings also have potential to be impactful beyond academia. Firstly, they serve as a basis for rejecting claims that British HE institutions are hotbeds of left‐wing bias. Taking steps to challenge such discourse is essential, as failure to do so will not only unduly tarnish the HE sector’s reputation—in ways which could impact funding and legitimacy—but may also heighten education‐based polarization in ways which could threaten societal cohesion and the functioning of democracy. Secondly, they indicate—contrary to assumptions made by those in politics and media—that generations of increasing HE enrollment rates, which mean the degree‐educated proportion of the British population rises by just under 1% point every year (Sobolewska & Ford, 2020), are unlikely to have any particularly dramatic effects in shifting aggregate British attitudes in the long‐term.

The limits of generalizability of this study’s findings must be taken seriously. If the HE effect is “different for people without a sibling,…sibling fixed‐effects models may over‐ or underestimate population‐level differences in…attitudes” engendered by university study (Campbell & Horowitz, 2016, p. 47). Given 45% of all UK families with dependent children have just one child (Office for National Statistics, 2015), the potential for bias here is not trivial. It should also be considered that this study’s results can only be generalized to families where some siblings attend HE and others do not as, by design, only siblings “who are discordant on exposure, contribute with statistical information to the estimation of education effects in… [within‐sibship] analysis” (Madsen et al., 2014, p. 188). As Western European societies are highly socially‐stratified environments in which educational attainment acts as an important status symbol (Bovens & Wille, 2017), it is possible that these types of families may be relatively rare, in Britain, or may differ from families where all siblings attend HE in important ways. Future research should therefore explore whether this study’s conclusion can be replicated using alternative quasi‐experimental study designs, which produce results generalizable to the entire graduate population.

To conclude, this study provides considerable evidence to suggest that within‐sibship designs produce less biased estimates of education’s causal effect on adult attitudes than conventional methods, which control only for measured confounders. The paper adds to a growing literature which demonstrates the value of quasi‐experimental methods in teasing out causal effects from observational data. More substantively, this paper offers novel insights in showing that obtaining a HE qualification only has a small *direct causal effect* on British individuals’ adult attitudes, and that this effect is not always liberalizing. Universities are not institutions of left‐liberal bias which encourage the development of distinctive political values. Rather, the well‐established association of HE with economic and cultural attitudes is largely spurious—materializing mostly because those who experience pre‐adult environments conducive to the formation of certain values disproportionately enroll in universities. Scholars should now expand the scope of this enquiry by using novel quasi‐experimental methods to identify how, and to what extent, educational attainment is causally linked with a range of adult outcomes.

## CONFLICT OF INTEREST

No conflict of interest to declare.

## ETHICS STATEMENT

Ethics approval for this study was granted by the University of Southampton Ethics Committee (ERGOII). Study number: 62220.

## Supporting information

Supporting Information S1

## Data Availability

The data that support the findings of this study are available from the UK Data Service. Restrictions apply to the availability of these data, which were used under license for this study. Data are available from https://beta.ukdataservice.ac.uk/datacatalogue/studies/study?id=6614 with the permission of the UK Data Service.

## ENDNOTES

^1^
Dey (1996), Paterson (2009, 2014), Stubager (2008) and Surridge (2016) suggest processes of (in)formal socialization experienced on educational campuses drive the direct education‐attitudes linkage.

^2^
Contrastingly, Van De Werfhorst and De Graaf (2004) find education is unrelated to economic preferences.

^3^
The median was used as the age at graduation distribution was strongly skewed right‐wards.

^4^
The economic attitudes scale is an outlier with a Cronbach’s Alpha (reliability test score) of 0.576, compared to >0.8 for the others. Guidelines suggest values of 0.6 are acceptable. It was therefore felt the economic attitudes scale was reliable enough for use.

^5^

^’^Missing’ categories were added to the psychological security and cognitive ability variables rather than excluding these from analysis, as doing so would engender omitted variable bias (see Appendix I) and raise theoretical questions, as these are important confounders. Imputation was not used as given the large volume of missing data it was feared even a small misspecification of the imputation model could cause large biases in the complete data. Imputation was therefore not believed to offer clear advantages over the “missing category” approach.

^6^
As the BHPS/Understanding Society data only contains birth year information for *respondents* in sampled households, birth‐order can only be classified among siblings who responded during the surveys’ 28 waves, rather than *all* siblings. While this measure may not always capture “true” birth‐order, it provides the best possible approximation, and nevertheless accounts for important birth‐order‐based differences in siblings’ experiences.

^7^
Only pre‐adult and parental attitudes corresponding with the attitudinal dependent variable are included.

^8^
Effect attenuation in within‐sibship analysis could also be driven by measurement error not shared by siblings (Frisell et al., 2012).

^9^
Paterson (2014) also uses the BHPS but does not report a full “with‐controls” HE effect.
