# Supplementary material for: Demystifying the link between higher education and liberal values: A within‐sibship analysis of British individuals’ attitudes from 1994–2020
Source: Br J Sociol. 2022 Aug 28;73(5):967–84. doi: 10.1111/1468-4446.12972 (PMC10087825; doi:10.1111/1468-4446.12972)
Supplement: Supplementary file 1 — Supporting Information S1 [file BJOS-73-967-s001.docx]

**Supplementary Materials for “Demystifying the Link Between Higher Education and Liberal Values: A Within-Sibship Analysis of British Individuals’ Attitudes from 1994-2020”**

**Appendix A:** Age Distribution of HE Graduates in the Full Combined Panel Sample

**Table A.A.1** – The Age Distribution of HE Graduates

| **Summary Statistic** | **Value** |
| --- | --- |
| 1^st^ percentile | 20 |
| 25^th^ percentile | 22 |
| 50^th^ percentile (median) | 23 |
| 75^th^ percentile | 27 |
| 99^th^ percentile | 62 |
| Mean | 27 |
| Standard deviation | 9.563 |
| Skewness | 2.169 |
| Kurtosis | 7.777 |

**Appendix B:** Comparison of Higher Education’s Reported Effect on Attitudes Based on Three and Four-Year Specifications

**Table A.B.1 -** The HE Effect on Gender Attitudes, 4- and 3- year specifications

| **Regression Model Statistics** | **(1.4) Education Only** | **(1.3) Education Only** | **(2.4) Self-selection** | **(2.3) Self-selection** | **(3.4) Self-selection + Pre-adult Attitudes** | **(3.3) Self-selection + Pre-adult Attitudes** | **(4.4) Sibling -Matched** | **(4.3) Sibling -Matched** |
| --- | --- | --- | --- | --- | --- | --- | --- | --- |
| Education coefficient – HE versus non-HE | 0.447*** | 0.426*** | 0.210*** | 0.193*** | 0.180*** | 0.176*** | 0.084 | 0.051 |
| Standard error | 0.022 | 0.021 | 0.038 | 0.038 | 0.037 | 0.036 | 0.065 | 0.066 |
| T value of coefficient | 20.61 | 20.63 | 5.51 | 5.15 | 4.81 | 4.91 | 1.29 | 0.077 |
| P value of coefficient | 0.000 | 0.000 | 0.000 | 0.000 | 0.000 | 0.000 | 0.199 | 0.439 |
| Observations | 10,775 | 11,048 | 2,242 | 2,296 | 2,027 | 2,171 | 1,151 | 1,278 |

Note: Estimates are based on complete cases and use sibling-custered standard errors. Significance is denoted by *** p < 0.01; ** p < 0.05; * p < 0.1.

**Table A.B.2 -** The HE Effect on Economic Attitudes, 4- and 3- year specifications

| **Regression Model Statistics** | **(1.4) Education Only** | **(1.3) Education Only** | **(2.4) Self-selection** | **(2.3) Self-selection** | **(3.4) Self-selection + Pre-adult Attitudes** | **(3.3) Self-selection + Pre-adult Attitudes** | **(4.4) Sibling -Matched** | **(4.3) Sibling -Matched** |
| --- | --- | --- | --- | --- | --- | --- | --- | --- |
| Education coefficient – HE versus non-HE | -0.134*** | -0.127*** | -0.069 | -0.070 | -0.073 | -0.050 | -0.022 | -0.011 |
| Standard error | 0.028 | 0.028 | 0.051 | 0.050 | 0.055 | 0.048 | 0.090 | 0.076 |
| T value of coefficient | -4.74 | 4.60 | -1.35 | -1.39 | -1.34 | -1.05 | -0.247 | -0.15 |
| P value of coefficient | 0.000 | 0.000 | 0.176 | 0.164 | 0.182 | 0.295 | 0.805 | 0.881 |
| Observations | 3,075 | 3,108 | 647 | 652 | 542 | 616 | 326 | 375 |

Note: Estimates are based on complete cases and use sibling-custered standard errors. Significance is denoted by *** p < 0.01; ** p < 0.05; * p < 0.1.

**Table A.B.3 -** The HE Effect on Environmental Attitudes, 4- and 3- year specifications

| **Regression Model Statistics** | **(1.4) Education Only** | **(1.3) Education Only** | **(2.4) Self-selection** | **(2.3) Self-selection** | **(3.4) Self-selection + Pre-adult Attitudes** | **(3.3) Self-selection + Pre-adult Attitudes** | **(4.4) Sibling -Matched** | **(4.3) Sibling -Matched** |
| --- | --- | --- | --- | --- | --- | --- | --- | --- |
| Education coefficient – HE versus non-HE | 0.402*** | 0.367*** | 0.184*** | 0.172*** | 0.122*** | 0.135*** | -0.045 | -0.118 |
| Standard error | 0.017 | 0.017 | 0.048 | 0.048 | 0.055 | 0.049 | 0.143 | 0.116 |
| T value of coefficient | 23.06 | 22.07 | 3.82 | 3.60 | 2.22 | 2.38 | -0.316 | -1.023 |
| P value of coefficient | 0.000 | 0.000 | 0.000 | 0.000 | 0.027 | 0.006 | 0.753 | 0.308 |
| Observations | 7,099 | 7,353 | 851 | 869 | 599 | 746 | 171 | 268 |

Note: Estimates are based on complete cases and use sibling-custered standard errors. Significance is denoted by *** p < 0.01; ** p < 0.05; * p < 0.1.

**Appendix C:** Comparison of the Distribution of Separate Understanding Society and BHPS Attitudinal Scales

**Table A.C.1 –** Summaries of Attitudinal Scales Constructed across Understanding Society and BHPS

| **Attitudinal Scale** | **Summary Statistics** | **Combined Summary Statistics** |
| --- | --- | --- |
| Gender attitudes | 1^st^ percentile – 1.0  25^th^ percentile – 2.7  50^th^ percentile (median) – 3.3  75^th^ percentile – 4.0  99^th^ percentile – 5.0  Mean – 3.2  Standard deviation – 0.9  Skewness – -0.1  Kurtosis – 2.6 |  |
| Economic attitudes | 1^st^ percentile – 1.8  25^th^ percentile – 3.0  50^th^ percentile (median) – 3.4  75^th^ percentile – 3.6  99^th^ percentile – 4.6  Mean – 3.3  Standard deviation – 0.6  Skewness – -0.2  Kurtosis – 3.2 |  |
| Environmentalism – Understanding Society | 1^st^ percentile – 1.9  25^th^ percentile – 2.9  50^th^ percentile (median) – 3.2  75^th^ percentile – 3.7  99^th^ percentile – 4.8  Mean – 3.3  Standard deviation – 0.6  Skewness – 0.1  Kurtosis – 3.0 | 1^st^ percentile – 1.9  25^th^ percentile – 2.9  50^th^ percentile (median) – 3.3  75^th^ percentile – 3.8  99^th^ percentile – 4.8  Mean – 3.3  Standard deviation – 0.6  Skewness – 0.1  Kurtosis – 2.9 |
| Environmentalism – BHPS | 1^st^ percentile – 2.0  25^th^ percentile – 3.0  50^th^ percentile (median) – 3.5  75^th^ percentile – 4.0  99^th^ percentile – 5.0  Mean – 3.5  Standard deviation – 0.6  Skewness – -0.2  Kurtosis – 2.9 |  |

Note: The three-year specification of HE graduation is used here.

**Appendix D:** Construction of Attitudinal Scales

**Table A.D.1** – Summary of Survey Items Included in Attitude Scales, and Reliability Measures of Resulting Scales

| **Attitude Measured** | **Survey Items Included** | **Cronbach’s Alpha** |
| --- | --- | --- |
| Gender roles | - A husband's job is to earn money, a wife's job is to look after the home and family. - All in all, family life suffers when the woman has a full-time job. - A pre-school child is likely to suffer if his or her mother works. | 0.801 |
| Economic Attitudes | - There is one law for the rich and one for the poor. - Private enterprise is the best way to solve the UK's economic problems - Major public services and industries ought to be in state ownership - It is the government's responsibility to provide a job for everyone who wants one. - Strong trade unions are needed to protect the working conditions and wages of employees | 0.576 |
| Environmentalism | **In Understanding Society:**   - My behaviour and everyday lifestyle contribute to climate change. - I would be prepared to pay more for environmentally-friendly products. - If things continue on their current course, we will soon experience a major environmental disaster. - The so-called 'environmental crisis' facing humanity has been greatly exaggerated. - Climate change is beyond control - it's too late to do anything about it. - The effects of climate change are too far in the future to really worry me. - Any changes I make to help the environment need to fit in with my lifestyle. - It's not worth me doing things to help the environment if others don't do the same. - It's not worth the UK trying to combat climate change, because other countries will just cancel out what we do.   **In the BHPS:**   - It takes too much time and effort to do things that are environmentally friendly - Scientists will find a solution to global warming without people having to make big changes to their lifestyle - The environment is a low priority for me compared with a lot of other things in my life - I am environmentally friendly in most things I do | **Understanding Society** = 0.993, **BHPS** = 0.970 |

Note: The three-year specification of HE graduation is used here.

**Appendix E:** Variable Coding and Summary Statistics

**Table A.E.1 –** Summary Statistics for All Variables Used in Analyses

| **Variable** | **Categories (if relevant)** | **Summary Statistic** |
| --- | --- | --- |
| Adult gender role attitude scale |  | 3.462 (0.899) |
| Adult economic attitude scale |  | 3.334 (0.547) |
| Adult environmentalism scale |  | 3.293 (0.613) |
| HE status | Graduate | 2,650 (4.42%) |
|  | Non-graduate | 57,292 (95.58%) |
| Gender | Male | 28,090 (46.86%) |
|  | Female | 31,852 (53.14%) |
| Cognitive ability | No information | 27,342 (45.61%) |
|  | Low ability | 12,387 (20.66%) |
|  | Medium ability | 11,117 (18.55%) |
|  | High ability | 9,096 (15.17%) |
| Psychological security | No information | 26,085 (43.52%) |
|  | Agree | 25,419 (42.41%) |
|  | Disagree | 8,438 (14.08%) |
| Occupational class | Managers & professionals | 3,121 (20.20%) |
|  | Intermediate | 2,605 (16.86%) |
|  | Semi(routine) | 5,180 (33.53%) |
|  | Inapplicable | 4,543 (29.41%) |
| Membership of community groups | Yes | 1,065 (1.78%) |
|  | No | 58,877 (98.22%) |
| Membership of sports groups | Yes | 2,612 (4.36%) |
|  | No | 57,330 (95.64%) |
| Participation in cultural activities | Yes | 4,384 (7.31%) |
|  | No | 55,558 (92.69%) |
| Birth order **(siblings only)** | Oldest sibling | 6,677 (48.62%) |
|  | Not the oldest | 7,057 (51.38%) |
| Pre-adult gender role attitude scale |  | 3.568 (0.845) |
| Pre-adult economic attitude scale |  | 3.316 (0.500) |
| Pre-adult environmentalism scale |  | 3.259 (0.592) |
| Parental occupation | Managers & professionals | 3,087 (28.38%) |
|  | Intermediate | 1,920 (17.65%) |
|  | Semi(routine) | 2,798 (25.72%) |
|  | Inapplicable | 3,074 (28.26%) |
| Parental education | Degree | 2,130 (19.78%) |
|  | Non-degree HE | 1,513 (14.05%) |
|  | A level, or equivalent | 1,844 (17.12%) |
|  | GCSE, or equivalent | 2,760 (25.63%) |
|  | Other qualifications | 1,136 (10.55%) |
|  | No qualifications | 1,385 (12.86%) |
| Parental income | Lowest quintile | 1,393 (12.75%) |
|  | 2^nd^ lowest | 1,988 (18.20%) |
|  | Middle | 2,553 (23.37%) |
|  | 2^nd^ highest | 2,858 (26.16%) |
|  | Highest quintile | 2,134 (19.53%) |
| Parental PTA membership | Yes | 571 (0.95%) |
|  | No | 59,371 (99.05%) |
| Parental gender role attitude scale |  | 3.300 (0.919) |
| Parental economic attitude scale |  | 3.398 (0.524) |
| Parental environmentalism scale |  | 3.355 (0.575) |

Note: Summary statistics reported are means with standard deviation in parentheses for continuous or scale variables. For categorical variables, each separate category is reported with the number of unique responses in each category and the proportion of the total variable this represents in parentheses. The three-year specification of HE graduation is used and all those with missing education are excluded.

**Appendix F:** Comparison of the Characteristics of the Full Combined Panel Sample, and the Sibling Only Panel Sample

**Table A.F.1 –** Comparing Composition of Full and Sibling Samples

| **Variable** | **Full Sample** | **Sibling Only Sample** |
| --- | --- | --- |
| Education: Degree in Panel | 4.42% | 12.36% |
| Education: No Degree | 95.58% | 87.64% |
| Gender: male | 46.86% | 51.52% |
| Gender: female | 53.14% | 48.48% |
| Class: managers & professionals | 20.20% | 19.48% |
| Class: intermediate | 16.86% | 16.10% |
| Class: semi(routine) | 33.53% | 35.56% |
| Class: inapplicable | 29.41% | 28.86% |
| Cognitive ability: low | 20.66% | 10.46% |
| Cognitive ability: medium | 18.55% | 13.46% |
| Cognitive ability: high | 15.17% | 13.46% |
| Cognitive ability: no information | 45.61% | 62.62% |
| What happens in life is beyond control: agree | 42.41% | 21.90% |
| What happens in life is beyond control: disagree | 14.08% | 11.58% |
| What happens in life is beyond control: no information | 43.52% | 66.52% |
| Community group participation: no | 98.22% | 93.64% |
| Community group participation: yes | 1.78% | 6.36% |
| Sport group participation: no | 95.64% | 84.35% |
| Sport group participation: yes | 4.36% | 15.65% |
| Cultural activity participation: no | 92.69% | 74.97% |
| Cultural activity participation: yes | 7.31% | 25.03% |

Note: This table shows the proportion of all unique responses in the full, and sibling, samples which fall into each of the categories represented here – it is designed to highlight any key differences between the composition of these samples. The three-year specification of HE graduation is used and all those with missing education are excluded.

As can be seen from Table A.F.1, the sibling sample are somewhat more educated, and more likely to have participated in cultural, sporting and community activities pre-adulthood, than the full sample. They are also somewhat more likely to have no information on the cognitive ability and psychological security questions.

**Appendix G:** Two-sample T-Tests for Differences in Pre-Adult and Adult Attitudes Reported by Educational Group

**Table A.G.1 –** Comparing Pre-adult and Adult Attitudes

|  | **Significant difference between graduates and non-graduates pre-university?** | **Significant difference between graduates and non-graduates post-university?** |
| --- | --- | --- |
| Gender attitudes | YES (p = 0.000) | YES (p = 0.000) |
| Economic attitudes | YES (p = 0.006) | YES (p = 0.000) |
| Environmentalism | YES (p = 0.000) | YES (p = 0.000) |

Note: The three-year specification of university duration is used.

**Appendix H:** Full Regression Results

**Table A.H.1** – Gender Attitudes Regression Results

|  | **(1) Education only** | **(2) Sibling education only model** | **(3) Self-Selection model** | **(4) Self-Selection and pre-adult attitudes model** | **(5) Sibling matched model** |
| --- | --- | --- | --- | --- | --- |
| HE status: Graduate | 0.426 *** | 0.338 *** | 0.193 *** | 0.176 *** | 0.051 |
|  | (0.021) | (0.039) | (0.038) | (0.036) | (0.065) |
| Gender: female |  |  | 0.267 *** | 0.118 *** | 0.074 |
|  |  |  | (0.034) | (0.033) | (0.056) |
| Cognitive ability: low |  |  | 0.076 | 0.119 ** | 0.119 |
|  |  |  | (0.054) | (0.052) | (0.093) |
| Cognitive ability: medium |  |  | 0.016 | 0.034 | 0.097 |
|  |  |  | (0.047) | (0.045) | (0.086) |
| Cognitive ability: high |  |  | 0.147 *** | 0.122 *** | 0.092 |
|  |  |  | (0.046) | (0.044) | (0.084) |
| Psychological security: disagree |  |  | 0.107 *** | 0.061 | 0.062 |
|  |  |  | (0.040) | (0.038) | (0.062) |
| Psychological security: no information |  |  | 0.124 *** | 0.102 ** | -0.131 * |
|  |  |  | (0.041) | (0.040) | (0.075) |
| Occupational class: intermediate |  |  | -0.100 * | -0.082 | -0.178 ** |
|  |  |  | (0.052) | (0.050) | (0.086) |
| Occupational class: (semi)routine |  |  | -0.098 ** | -0.094 ** | -0.092 |
|  |  |  | (0.045) | (0.043) | (0.074) |
| Occupational class: inapplicable |  |  | -0.215 *** | -0.153 *** | -0.102 |
|  |  |  | (0.049) | (0.047) | (0.084) |
| Membership of community groups: yes |  |  | 0.010 | 0.027 | 0.019 |
|  |  |  | (0.052) | (0.049) | (0.088) |
| Membership of sport groups: yes |  |  | -0.011 | 0.019 | 0.066 |
|  |  |  | (0.036) | (0.034) | (0.064) |
| Participation in cultural activities: yes |  |  | 0.079 ** | 0.054 | -0.017 |
|  |  |  | (0.035) | (0.034) | (0.067) |
| Parental occupation: intermediate |  |  | 0.078 | 0.078 |  |
|  |  |  | (0.052) | (0.050) |  |
| Parental occupation: (semi)routine |  |  | 0.017 | 0.022 |  |
|  |  |  | (0.052) | (0.050) |  |
| Parental occupation: inapplicable |  |  | -0.038 | -0.010 |  |
|  |  |  | (0.061) | (0.058) |  |
| Parental education: non-degree HE |  |  | -0.098 | -0.086 |  |
|  |  |  | (0.063) | (0.061) |  |
| Parental education: A level, or equiv. |  |  | -0.112 * | -0.098 |  |
|  |  |  | (0.063) | (0.061) |  |
| Parental education: GCSE, or equiv. |  |  | -0.048 | -0.049 |  |
|  |  |  | (0.059) | (0.056) |  |
| Parental education: other quals |  |  | -0.163 ** | -0.131 * |  |
|  |  |  | (0.072) | (0.069) |  |
| Parental education: no quals |  |  | -0.078 | -0.042 |  |
|  |  |  | (0.069) | (0.066) |  |
| Parental income: 2^nd^ lowest |  |  | 0.083 | 0.096 |  |
|  |  |  | (0.065) | (0.062) |  |
| Parental income: middle |  |  | 0.127 ** | 0.134 ** |  |
|  |  |  | (0.064) | (0.061) |  |
| Parental income: 2^nd^ highest |  |  | 0.133 ** | 0.124 * |  |
|  |  |  | (0.067) | (0.064) |  |
| Parental income: highest |  |  | 0.187 ** | 0.167 ** |  |
|  |  |  | (0.076) | (0.073) |  |
| Parental PTA membership: yes |  |  | -0.081 | -0.054 |  |
|  |  |  | (0.063) | (0.060) |  |
| Parental gender attitude |  |  | 0.187 *** | 0.103 *** |  |
|  |  |  | (0.019) | (0.019) |  |
| Pre-adult gender attitude |  |  |  | 0.380 *** | 0.300 *** |
|  |  |  |  | (0.021) | (0.037) |
| **Intercept** | 3.383 *** | 3.463 *** | 2.781 *** | 1.749 *** | 1.887 *** |
|  | (0.009) | (0.022) | (0.117) | (0.128) | (0.500) |
| **Birth order (oldest versus other)** |  |  |  |  | -0.081 * |
|  |  |  |  |  | (0.043) |
| **Sibling fixed effects** | NO | NO | NO | NO | YES |
| **Observations** | 11048 | 2240 | 2296 | 2171 | 1278 |
| **R2** | 0.034 | 0.033 | 0.147 | 0.254 | 0.644 |

Note: regression coefficients are presented with sibling-clustered standard errors in parentheses. Significance is denoted by *** p < 0.01; ** p < 0.05; * p < 0.1.

**Table A.H.2** – Economic Attitudes Regression Results

|  | **(1) Education only** | **(2) Sibling education only model** | **(3) Self-Selection model** | **(4) Self-Selection and pre-adult attitudes model** | **(5) Sibling matched model** |
| --- | --- | --- | --- | --- | --- |
| HE status: Graduate | -0.127 *** | -0.058 | -0.070 | -0.050 | -0.011 |
|  | (0.028) | (0.046) | (0.050) | (0.048) | (0.076) |
| Gender: female |  |  | 0.048 | 0.021 | 0.028 |
|  |  |  | (0.040) | (0.037) | (0.062) |
| Cognitive ability: low |  |  | 0.013 | 0.015 | -0.041 |
|  |  |  | (0.068) | (0.064) | (0.101) |
| Cognitive ability: medium |  |  | -0.014 | -0.005 | -0.011 |
|  |  |  | (0.057) | (0.053) | (0.100) |
| Cognitive ability: high |  |  | -0.065 | -0.073 | 0.053 |
|  |  |  | (0.055) | (0.052) | (0.098) |
| Psychological security: disagree |  |  | -0.005 | 0.043 | -0.051 |
|  |  |  | (0.044) | (0.041) | (0.064) |
| Psychological security: no information |  |  | -0.036 | 0.024 | 0.006 |
|  |  |  | (0.054) | (0.050) | (0.088) |
| Occupational class: intermediate |  |  | 0.158 *** | 0.108 ** | -0.081 |
|  |  |  | (0.059) | (0.055) | (0.096) |
| Occupational class: (semi)routine |  |  | 0.155 *** | 0.122 ** | 0.102 |
|  |  |  | (0.054) | (0.050) | (0.083) |
| Occupational class: inapplicable |  |  | 0.176 *** | 0.145 ** | 0.138 |
|  |  |  | (0.061) | (0.057) | (0.102) |
| Membership of community groups: yes |  |  | 0.074 | 0.093 | 0.291 *** |
|  |  |  | (0.062) | (0.057) | (0.109) |
| Membership of sport groups: yes |  |  | 0.014 | 0.005 | 0.036 |
|  |  |  | (0.040) | (0.037) | (0.065) |
| Participation in cultural activities: yes |  |  | 0.002 | 0.014 | 0.010 |
|  |  |  | (0.041) | (0.038) | (0.072) |
| Parental occupation: intermediate |  |  | 0.031 | 0.028 |  |
|  |  |  | (0.064) | (0.059) |  |
| Parental occupation: (semi)routine |  |  | -0.032 | -0.011 |  |
|  |  |  | (0.063) | (0.058) |  |
| Parental occupation: inapplicable |  |  | 0.014 | 0.032 |  |
|  |  |  | (0.074) | (0.069) |  |
| Parental education: non-degree HE |  |  | 0.165 * | 0.221 *** |  |
|  |  |  | (0.089) | (0.083) |  |
| Parental education: A level, or equiv. |  |  | 0.185 * | 0.163 * |  |
|  |  |  | (0.094) | (0.089) |  |
| Parental education: GCSE, or equiv. |  |  | 0.207 ** | 0.191 ** |  |
|  |  |  | (0.086) | (0.080) |  |
| Parental education: other quals |  |  | 0.093 | 0.133 |  |
|  |  |  | (0.101) | (0.094) |  |
| Parental education: no quals |  |  | 0.278 *** | 0.260 *** |  |
|  |  |  | (0.095) | (0.089) |  |
| Parental income: 2^nd^ lowest |  |  | 0.024 | 0.083 |  |
|  |  |  | (0.070) | (0.065) |  |
| Parental income: middle |  |  | 0.042 | 0.096 |  |
|  |  |  | (0.071) | (0.066) |  |
| Parental income: 2^nd^ highest |  |  | 0.001 | 0.006 |  |
|  |  |  | (0.077) | (0.072) |  |
| Parental income: highest |  |  | 0.090 | 0.066 |  |
|  |  |  | (0.088) | (0.081) |  |
| Parental PTA membership: yes |  |  | 0.049 | 0.039 |  |
|  |  |  | (0.067) | (0.063) |  |
| Parental economic attitude |  |  | 0.136 *** | 0.015 |  |
|  |  |  | (0.036) | (0.035) |  |
| Pre-adult economic attitude |  |  |  | 0.442 *** | 0.485 *** |
|  |  |  |  | (0.038) | (0.064) |
| **Intercept** | 3.353 *** | 3.341 *** | 2.517 *** | 1.431 *** | 1.819 *** |
|  | (0.011) | (0.023) | (0.167) | (0.181) | (0.362) |
| **Birth order (oldest versus other)** |  |  |  |  | 0.034 |
|  |  |  |  |  | (0.049) |
| **Sibling fixed effect** | NO | NO | NO | NO | YES |
| **Observations** | 3108 | 569 | 652 | 616 | 375 |
| **R2** | 0.007 | 0.003 | 0.095 | 0.270 | 0.661 |

Note: Regression coefficients are presented with sibling-clustered standard errors in parentheses. Significance is denoted by *** p < 0.01; ** p < 0.05; * p < 0.1.

**Table A.H.3** – Environmental Attitudes Regression Results

|  | **(1) Education only** | **(2) Sibling education only model** | **(3) Self-Selection model** | **(4) Self-Selection and pre-adult attitudes model** | **(5) Sibling matched model** |
| --- | --- | --- | --- | --- | --- |
| HE status: Graduate | 0.367 *** | 0.315 *** | 0.172 *** | 0.135 *** | -0.118 |
|  | (0.017) | (0.032) | (0.048) | (0.049) | (0.116) |
| Gender: female |  |  | 0.131 *** | 0.066 | 0.035 |
|  |  |  | (0.043) | (0.044) | (0.091) |
| Cognitive ability: low |  |  | -0.153 ** | -0.129 * | 0.071 |
|  |  |  | (0.068) | (0.069) | (0.145) |
| Cognitive ability: medium |  |  | -0.098 | -0.037 | 0.196 |
|  |  |  | (0.061) | (0.062) | (0.154) |
| Cognitive ability: high |  |  | 0.015 | 0.039 | 0.264 ** |
|  |  |  | (0.060) | (0.061) | (0.132) |
| Psychological security: disagree |  |  | 0.001 | 0.015 | 0.041 |
|  |  |  | (0.061) | (0.063) | (0.128) |
| Psychological security: no information |  |  | 0.124 ** | 0.164 *** | 0.043 |
|  |  |  | (0.050) | (0.050) | (0.124) |
| Occupational class: intermediate |  |  | -0.024 | -0.041 | -0.551 *** |
|  |  |  | (0.068) | (0.071) | (0.160) |
| Occupational class: (semi)routine |  |  | -0.011 | 0.002 | -0.214 * |
|  |  |  | (0.057) | (0.057) | (0.127) |
| Occupational class: inapplicable |  |  | 0.052 | 0.045 | -0.187 |
|  |  |  | (0.060) | (0.061) | (0.145) |
| Membership of community groups: yes |  |  | 0.140 ** | 0.083 | 0.201 |
|  |  |  | (0.065) | (0.064) | (0.137) |
| Membership of sport groups: yes |  |  | 0.005 | 0.004 | 0.032 |
|  |  |  | (0.048) | (0.048) | (0.116) |
| Participation in cultural activities: yes |  |  | 0.089 * | 0.053 | -0.010 |
|  |  |  | (0.046) | (0.048) | (0.112) |
| Parental occupation: intermediate |  |  | -0.042 | -0.046 |  |
|  |  |  | (0.066) | (0.067) |  |
| Parental occupation: (semi)routine |  |  | -0.068 | -0.057 |  |
|  |  |  | (0.065) | (0.065) |  |
| Parental occupation: inapplicable |  |  | -0.127 * | -0.150 * |  |
|  |  |  | (0.077) | (0.080) |  |
| Parental education: non-degree HE |  |  | -0.161 ** | -0.114 |  |
|  |  |  | (0.070) | (0.072) |  |
| Parental education: A level, or equiv. |  |  | -0.174 ** | -0.089 |  |
|  |  |  | (0.074) | (0.075) |  |
| Parental education: GCSE, or equiv. |  |  | -0.169 ** | -0.126 * |  |
|  |  |  | (0.070) | (0.071) |  |
| Parental education: other quals |  |  | -0.148 * | -0.132 |  |
|  |  |  | (0.088) | (0.089) |  |
| Parental education: no quals |  |  | -0.303 *** | -0.188 ** |  |
|  |  |  | (0.095) | (0.095) |  |
| Parental income: 2^nd^ lowest |  |  | -0.009 | 0.064 |  |
|  |  |  | (0.089) | (0.089) |  |
| Parental income: middle |  |  | -0.022 | 0.024 |  |
|  |  |  | (0.086) | (0.086) |  |
| Parental income: 2^nd^ highest |  |  | -0.105 | -0.096 |  |
|  |  |  | (0.087) | (0.088) |  |
| Parental income: highest |  |  | -0.082 | -0.019 |  |
|  |  |  | (0.096) | (0.097) |  |
| Parental PTA membership: yes |  |  | -0.095 | -0.116 |  |
|  |  |  | (0.089) | (0.091) |  |
| Parental environmental attitude |  |  | 0.079 ** | -0.021 |  |
|  |  |  | (0.037) | (0.038) |  |
| Pre-adult environmental attitude |  |  |  | 0.349 *** | 0.091 |
|  |  |  |  | (0.038) | (0.086) |
| **Intercept** | 3.199 *** | 3.241 *** | 3.182 *** | 2.341 *** | 2.701 *** |
|  | (0.008) | (0.018) | (0.184) | (0.208) | (0.500) |
| **Birth order (oldest versus other)** |  |  |  |  | -0.081 |
|  |  |  |  |  | (0.080) |
| **Sibling fixed effect** | NO | NO | NO | NO | YES |
| **Observations** | 7353 | 1769 | 869 | 746 | 268 |
| **R2** | 0.068 | 0.056 | 0.143 | 0.239 | 0.726 |

Note: regression coefficients are presented with sibling-clustered standard errors in parentheses. Significance is denoted by *** p < 0.01; ** p < 0.05; * p < 0.1.

**Appendix I:** Sensitivity Analysis for the In/Exclusion of Psychological Security and Cognitive Ability Variables

**Table A.I.1 – Models Including and Excluding Psychological Security**

|  | **(3) Self-Selection** | **(3) MINUS PSYCH. SECURITY** | **(4) Self-Selection and pre-adult attitudes** | **(4) MINUS PSYCH. SECURITY** | **(5) Sibling -matched** | **(5) MINUS PSYCH. SECURITY** |
| --- | --- | --- | --- | --- | --- | --- |
| **Gender Attitudes** | | | | | | |
| HE status: Graduate | 0.193*** | 0.202*** | 0.176*** | 0.184*** | 0.051 | 0.055 |
|  | (0.038) | (0.037) | (0.036) | (0.036) | (0.066) | (0.066) |
| Disagree | 0.107*** (0.040) |  | 0.061 (0.037) |  | 0.062 (0.063) |  |
| No info | 0.124*** (0.042) |  | 0.102** (0.041) |  | -0.131 (0.082) |  |
| Observations | 2,296 | | 2,171 | | 1,278 | |
| **Economic Attitudes** | | | | | | |
| HE status: Graduate | -0.070 | -0.071 | -0.050 | -0.048 | -0.011 | -0.013 |
|  | (0.050) | (0.050) | (0.048) | (0.048) | (0.076) | (0.074) |
| Disagree | -0.005 (0.043) |  | 0.043 (0.040) |  | -0.051 (0.066) |  |
| No info | -0.036 (0.053) |  | 0.024 (0.048) |  | 0.006 (0.083) |  |
| Observations | 652 | | 616 | | 375 | |
| **Environmental Attitudes** | | | | | | |
| HE status: Graduate | 0.172*** | 0.177*** | 0.135*** | 0.152*** | -0.118 | -0.116 |
|  | (0.048) | (0.048) | (0.049) | (0.049) | (0.116) | (0.110) |
| Disagree | 0.001 (0.062) |  | 0.015 (0.066) |  | 0.041 (0.128) |  |
| No info | 0.124** (0.051) |  | 0.164 (0.053) |  | 0.043 (0.106) |  |
| Observations | 869 | | 746 | | 268 | |

Note: regression coefficients are presented with sibling-clustered standard errors in parentheses. Significance is denoted by *** p < 0.01; ** p < 0.05; * p < 0.1.

At least one category of psychological security is statistically significant (at the 5% threshold) in 3 of the 9 attitudinal models. Failing to include this variable also leads to changes (generally increases) in the size of HEs effect on attitudes – this shows that, in most cases, psychological security is working as a control variable, as expected. Omitted variable bias is engendered if this variable is left out of analysis.

**Table A.I.2 – Models Including and Excluding Cognitive Ability**

|  | **(3) Self-Selection** | **(3) MINUS COG. ABILITY** | **(4) Self-Selection and pre-adult attitudes** | **(4) MINUS COG. ABILITY** | **(5) Sibling - matched** | **(5) MINUS COG. ABILITY** |
| --- | --- | --- | --- | --- | --- | --- |
| **Gender Attitudes** | | | | | | |
| HE status: Graduate | 0.193*** | 0.208*** | 0.176*** | 0.181*** | 0.051 | 0.055 |
|  | (0.038) | (0.037) | (0.036) | (0.035) | (0.066) | (0.064) |
| Low ability | 0.076 (0.055) |  | 0.119** (0.055) |  | 0.119 (0.100) |  |
| Med. ability | 0.016 (0.048) |  | 0.034 (0.046) |  | 0.097 (0.092) |  |
| High ability | 0.147*** (0.047) |  | 0.122*** (0.045) |  | 0.092 (0.088) |  |
| Observations | 2,296 | | 2,171 | | 1,278 | |
| **Economic Attitudes** | | | | | | |
| HE status: Graduate | -0.070 | -0.084* | -0.050 | -0.064 | -0.011 | 0.002 |
|  | (0.050) | (0.049) | (0.048) | (0.046) | (0.076) | (0.076) |
| Low ability | 0.013 (0.062) |  | 0.015 (0.057) |  | -0.041 (0.084) |  |
| Med. ability | -0.014 (0.052) |  | -0.005 (0.047) |  | -0.011 (0.087) |  |
| High ability | -0.065 (0.053) |  | -0.073 (0.050) |  | 0.053 (0.082) |  |
| Observations | 652 | | 616 | | 375 | |
| **Environmental Attitudes** | | | | | | |
| HE status: Graduate | 0.172*** | 0.199*** | 0.135*** | 0.154*** | -0.118 | -0.106 |
|  | (0.048) | (0.047) | (0.049) | (0.048) | (0.116) | (0.119) |
| Low ability | -0.153** (0.067) |  | -0.129* (0.070) |  | 0.071 (0.158) |  |
| Med. ability | -0.098 (0.061) |  | -0.037 (0.065) |  | 0.196 (0.146) |  |
| High ability | 0.015 (0.061) |  | 0.039 (0.064) |  | 0.264** (0.113) |  |
| Observations | 869 | | 746 | | 268 | |

Note: regression coefficients are presented with sibling-clustered standard errors in parentheses. Significance is denoted by *** p < 0.01; ** p < 0.05; * p < 0.1.

At least one category of cognitive ability is statistically significant (at the 5% threshold) in 4 of the 9 attitudinal models. Failing to include this variable also leads to changes (generally increases) in the size of HEs effect on attitudes. Omitted variable bias is engendered if this variable is left out of analysis.
